# Surgical Outcomes of Levator Resection in Moderate and Severe Ptosis With a Good Levator Function: A Retrospective Study

**DOI:** 10.7759/cureus.42136

**Published:** 2023-07-19

**Authors:** Sucharita Das, Anupam Singh, Srishti Sharma, Mittali Khurana, Pooja Kumari, Sanjeev K Mittal, Barun Kumar

**Affiliations:** 1 Ophthalmology, All India Institute of Medical Sciences, Rishikesh, Rishikesh, IND; 2 Cardiology, All India Institute of Medical Sciences, Rishikesh, Rishikesh, IND

**Keywords:** good to fair levator function., moderate to severe ptosis, congenital simple ptosis, ptosis, levator resection

## Abstract

Introduction: An abnormally drooping upper eyelid in comparison with the normal position in primary gaze refers to ptosis. Levator resection should be the procedure of choice in patients with moderate to severe ptosis and a good levator function.

Methods: In this retrospective study, we analysed the surgical outcomes after large and maximal levator resection in patients with moderate and severe ptosis with a good levator function and Bell’s phenomenon. All patients had a good levator function; therefore, levator resection was the procedure of choice. We performed levator resection of 20 mm and above and the desired post-operative correction was achieved.

Results: One patient had microcornea, and hence, he was undercorrected and his post-operative marginal reflex distance 1 (MRD 1) was 3 mm. Two patients who had severe ptosis pre-operatively had a post-operative MRD 1 of 3 mm. Rest of the patients had a post-operative MRD 1 of 4 mm.

Conclusion: Levator resection of 20 mm or more should be performed in patients with congenital simple ptosis with a good levator function and Bell's phenomenon to achieve a favourable post-operative outcome.

## Introduction

An abnormally drooping upper eyelid in comparison with the normal position in primary gaze refers to ptosis. Ptosis is classified as congenital if it is noticed at birth or in infancy [1,2]. Acquired ptosis results due to aponeurotic, myogenic, neurogenic, or mechanical causes. Various surgical options include levator muscle resection, frontalis sling, Fasanella-Servat procedure, and Müller's muscle-conjunctival resection [3]. Levator resection creates optimal lid contour and symmetry that is a desirable cosmetic outcome [4]. In transcutaneous levator resection, the muscle must be detached from the underlying conjunctiva and reattached to the mid-tarsal plate until the eyelid margin reaches the desired height [5]. Levator resection should be the procedure of choice in patients with moderate to severe ptosis and a good levator function [5-7]. Nirankari et al. suggested resection should be 3 mm for every 1 mm of ptosis in patients with a good levator function, with a basic increment of 5 mm to make up for the minimum of 8 mm resection. For instance, 1 mm of ptosis would require 3 mm + 5 mm of resection, or 8 mm; 2 mm of ptosis would require 6 mm plus 5 mm, or 11 mm; and so on [8]. Beard suggested the use of degree of ptosis and the grade of levator palpebrae superioris (LPS) function to calculate the pre-operative amount of resection required [9-11]. We present this study to report the surgical outcomes after large and maximal levator resection in moderate and severe ptosis cases with a good levator function and Bell’s phenomenon.

## Materials and methods

This retrospective study, conducted at All India Institute of Medical Sciences, Rishikesh, included 10 consecutive patients of congenital simple ptosis who underwent levator resection (from January 2022 to December 2022) for moderate and severe ptosis with good LPS action and Bell’s phenomenon with a minimum follow-up of six months. The study adhered to the Declaration of Helsinki and an ethical approval was obtained from the Institutional Ethics Committee (letter no. AIIMS/IEC.23/127 dated April 15, 2023); written informed consent was obtained from all participants.

**Figure 1:**
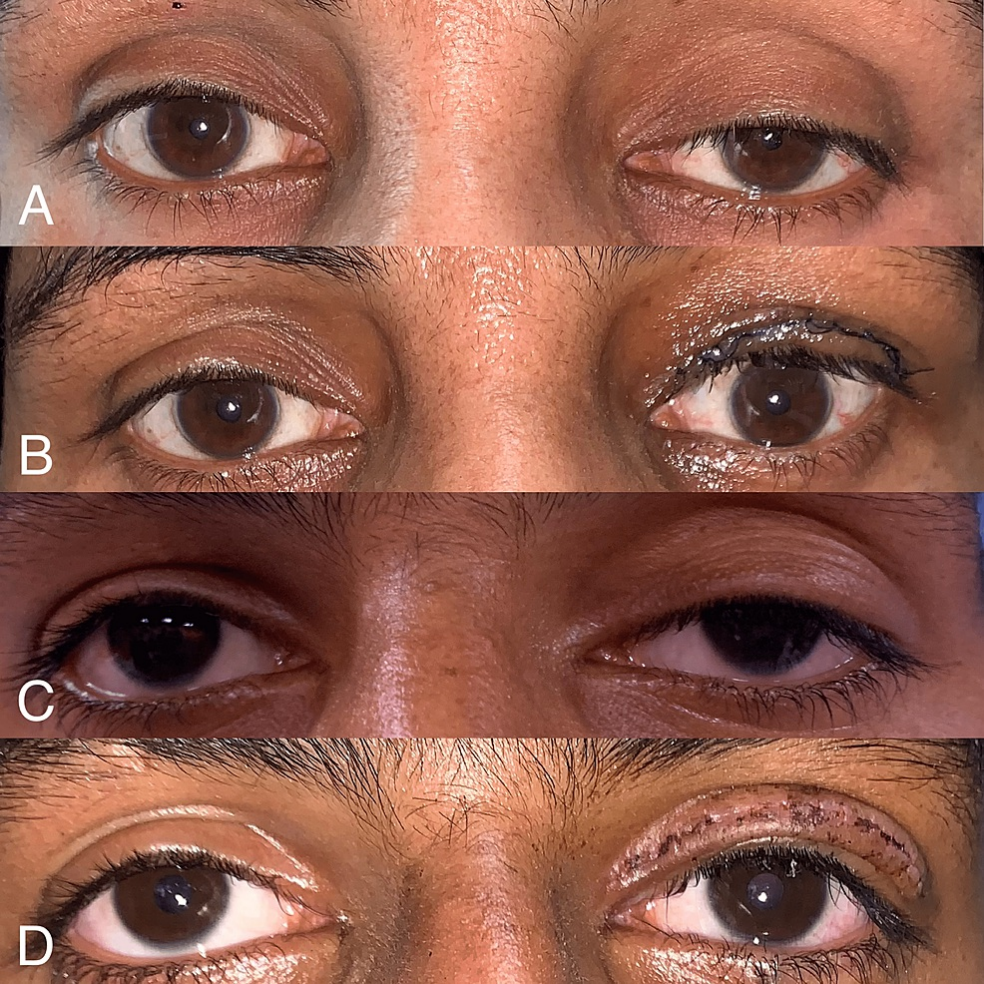
Pre- and post-operative clinical photographs of two patients MRD 1, marginal reflex distance 1 (A) Pre-operative clinical photograph of the left eye exhibiting mild ptosis (Patient 4). (B) Post-operative clinical photograph of the left eye showing improvement in MRD 1 (Patient 4). (C) Pre-operative clinical photograph of the left eye exhibiting severe ptosis (Patient 10). (D) Post-operative clinical photograph of the left eye showing improvement in MRD 1 (Patient 10).

All patients underwent a proper, basic ophthalmic examination including cycloplegic refraction, orthoptic examination, and detailed anterior and posterior segment examination by the slit-lamp biomicroscopy. Patients having acquired, aponeurotic, complicated, neurogenic ptosis, any etiology other than congenital ptosis, or having poor levator action or poor Bell’s phenomenon, dry eye disorder, not willing for surgical intervention, and with a follow-up of less than six months were excluded from the study. We followed the recommendation by Nirankari et al. and Beard for calculating the amount of levator resection (Table 1) [8-11]. Any value of the marginal reflex distance 1 (MRD 1) within 1 mm of the normal reading (4 mm) was considered as a successful/favourable outcome. We performed levator resection of 20 mm and above and the post-operative correction of desired height was achieved. Patients were followed up for a minimum period of six months. Pre- and post-operative clinical photographs of two patients are shown in Figure 1.

**Table 1:** Amount of levator resection based on Beard’s pre-operative evaluation Ref. [9]

Degree of ptosis	Levator function	Resection
Mild (<2 mm)	>10 mm	Small (10-13 mm)
Moderate (3 mm)	>8 mm	Moderate (14-17 mm)
Moderate (3 mm)	5-8 mm	Large (18-22 mm)
Severe (>4 mm)	<5 mm	Maximum (>23 mm)

## Results

The baseline characteristics of the patients are summarized in Table 2. There were two males and eight females with a mean age of 23.2 years. All the patients had simple congenital ptosis. A total of 90% patients had a good levator function, and one patient had a fair levator function. One patient had microcornea, and hence, he was undercorrected; his post-operative MRD 1 was 3 mm. Two patients who had severe ptosis pre-operatively had a post-operative MRD 1 of 3 mm. Rest of the patients had a post-operative MRD 1 of 4 mm. Thus, all the patients had a favorable outcome. Post-operative lid edema and lagophthalmos were observed in all cases and one patient had mild overcorrection (with a post-operative MRD 1 of 5 mm) that resolved in the subsequent follow-up with lid massage and did not require surgical intervention.

**Table 2:** Amount of levator resection and post-operative MRD 1 MRD 1, marginal reflex distance 1

Patient no.	Age/gender	Etiology	Pre-operative MRD 1	Levator function	Amount of levator resection	Post-operative MRD 1
1	29/F	Simple congenital	2 mm	10 mm	22 mm	4 mm
2	23/M	Simple congenital	1 mm	10 mm	20 mm	3 mm
3	35/F	Simple congenital	2 mm	12 mm	22 mm	4 mm
4	25/F	Simple congenital	2 mm	12 mm	22 mm	4 mm
5	31/F	Simple congenital	0 mm	10 mm	24 mm	5 mm
6	26/F	Simple congenital	2 mm	10 mm	22 mm	4 mm
7	16/F	Simple congenital	2 mm	12 mm	22 mm	4 mm
8	16/F	Simple congenital	2 mm	12 mm	22 mm	4 mm
9	15/F	Simple congenital	-1 mm	8 mm	24 mm	3 mm
10	16/M	Simple congenital	0 mm	10 mm	22 mm	3 mm

## Discussion

It is generally agreed upon that patients with a levator function of more than 4 mm should have levator resection surgery [12]. As our patients had a good levator function, levator resection was the choice of surgery. To achieve a proper eyelid contour and position during levator resection, a thorough understanding of eyelid anatomy is necessary. Furthermore, the procedure takes a long time, necessitates dissecting various structures of the eyelid, and involves patient cooperation [13]. The choice of surgery depends on both the extent of levator function and the amount of eyelid ptosis. Resection is often employed for treating ptosis with good levator excursion (8-12 mm) [5]. The benefit of this method includes conservation of all elevating components, including Müller's muscle and Whitnall's ligament, as well as the normal anatomical planes and structures of the eyelid [1]. The degree of ptosis and the grade of LPS function are used for the pre-operative calculation of the required amount of resection. When levator muscle activity is poor to fair, the lid margin is positioned at the superior corneal limbus, and a subsequent drop of several millimetres is anticipated. When the lid is positioned 2-3 mm below the limbus with a fair to good function, no post-operative drop is anticipated [14]. Levator excision was performed by Cates and Tyers on 100 patients who were younger than seven years old and had congenital ptosis with a levator function of at least 4 mm [15]. A post-operative lid margin position that was within 1 mm of normal was considered a success in their report in 75% of cases. Undercorrection was shown to be the most frequent issue in that study (19%), while overcorrection was far less frequent (7%). They discovered that the best indicator of a successful outcome from levator resection surgery was the level of levator function present before the operation.

According to Nuhoglu et al., patients with a poor levator function experienced undercorrection more frequently. The amount of levator function, however, was noticeably higher in the overcorrected group [16]. In our study, we observed that a post-operative satisfactory result was obtained with levator resection of 20 mm and above for a good levator function in moderate ptosis cases. As seen in the study by Wuthisiri et al., the mean length of the levator muscle that was resected was the only parameter that had any significant impact on the surgical outcome. In the successful and unsuccessful groups, the mean lengths were 18.15 ± 0.44 mm and 14.29 ± 0.94 mm (p = 0.011), respectively. Lagophthalmos (81.58%) was one of the surgical consequences, and it was discovered that the severity of the condition and ocular surface exposure eventually improved over time [17]. In our study, all the cases had lagophthalmos in the immediate post-operative period and resolved on subsequent follow-up visits within six weeks.

Limitations

Our study also had some limitations that must be mentioned. Firstly, this was a retrospective study; therefore, recall bias and interviewer bias cannot be ruled out. Furthermore, the sample size was small with only 10 patients. Also, the follow-up duration was only of six months, which is another limitation.

## Conclusions

Congenital simple ptosis is one of the most common types of ptosis we encounter in clinics. Levator resection is the procedure of choice in cases of congenital simple ptosis with a good levator function and Bell's phenomenon. There are various methods for the calculation of amount of levator resection in such cases. Through this study, we conclude that irrespective of the other factors, levator resection of 20 mm or more should be performed in these cases to get a favourable post-operative outcome.
